# Increased genetic variation of bovine viral diarrhea virus in dairy cattle in Poland

**DOI:** 10.1186/s12917-019-2029-z

**Published:** 2019-08-05

**Authors:** Paweł Mirosław, Mirosław Polak

**Affiliations:** Department of Virology of the National Veterinary Research Institute, Partyzantów 57, 24-100 Puławy, Poland

**Keywords:** Bovine viral diarrhea virus, Genetic diversity, Subtypes, Pestivirus, Cattle

## Abstract

**Background:**

Bovine viral diarrhea virus (BVDV) causes severe economic losses and is one of the most important viral pathogens of ruminants worldwide. The infection manifests itself in a variety of clinical symptoms. Phylogenetic studies based mainly on 5’UTR of its genome, identified many different subtypes of BVDV. Previous study indicated the predominance of BVDV-1b and BVDV-1d in Poland. The aim of this study was to genotype BVDV isolates currently circulating in Polish dairy herds.

**Results:**

BVDV was detected in 30 herds. Viral subtypes were identified using sequences of the 5’UTR fragment and they were confirmed within a fragment of the N^pro^ region. Seven subtypes of BVDV-1 species have been identified: 1b, 1 g, 1f, 1d, 1r, 1 s and 1e.

**Conclusion:**

The number of subtypes of BVDV in Poland evolves and 2 new subtypes have been identified for the first time. Such studies may have a positive impact on successful eradication of the virus using effective vaccines and diagnostic tests.

**Electronic supplementary material:**

The online version of this article (10.1186/s12917-019-2029-z) contains supplementary material, which is available to authorized users.

## Background

Bovine viral diarrhea virus (BVDV) belongs to *Pestivirus* genus in the *Flaviviridae* family [1]. It consists of four recognized species: bovine viral diarrhea virus type 1 (BVDV-1, Pestivirus A), type 2 (BVDV-2, Pestivirus B), classical swine fever virus (CSFV, Pestivirus C) and border disease virus (BDV, Pestivirus D). A few putative species have been discovered recently which may be classified as members of the *Pestivirus* genus but they have not been approved as species yet. Among them are: HoBi-like pestiviruses (also called BVDV-3) identified first in batches of contaminated foetal calf serum [2] and then in calves and aborted fetuses [3, 4], giraffe pestivirus associated with the outbreak of mucosal-like disease in Kenyan giraffes [5], Bungowannah virus detected in pig herds in Australia where stillbirth foetuses and neonatal deaths were observed [6] and Pronghorn virus, isolated from a pronghorn antelope in the United States [7]. There are also reports of novel pestiviruses in other animal species like rats and bats [8, 9]. This wide range of pestiviruses infecting different animal species is the proof of genetic plasticity of their genomes, adapting to different hosts.

BVDV is an important pathogen of cattle worldwide with significant economic impact [10]. Infection may lead to a wide array of clinical signs from subclinical to severe acute hemorrhagic syndrome and fatal mucosal disease [11]. BVDV also causes immunosuppression, which increases the severity of clinical picture when other pathogens are involved. BVDV infection of seronegative and pregnant females during the first 40–120 days of pregnancy may lead to the birth of persistently infected (PI) calves. They remain infected for life and shed the virus in high titre, ensuring the persistence of BVDV in the herd if they are not removed immediately after identification.

Viral genome is comprised of a single-stranded positive sense RNA about 12.3 kb in size with one large open reading frame flanked by 5′ and 3′ untranslated regions (5’UTR and 3’UTR respectively) [1]. Pestiviral genome encodes a single polyprotein that is processed into either 11 or 12 proteins: N^pro^, C, E^rns^, E1, E2, p7, NS2–3 (NS2, NS3), NS4A, NS4B, NS5A, NS5B. Several regions of BVDV genome have been used to study its genetic diversity [12, 13]. Phylogenetic analysis is mostly based on the comparison of nucleotide sequences from the 5’UTR, N^pro^ or E2 regions of viral genome. Based on genetic studies, 21 subtypes of BVDV-1 (1a - 1u) and 4 subtypes of BVDV-2 (2a – 2d) were identified so far [14, 15]. BVDV-1 is the predominant pestivirus circulating in cattle population in Europe [16]. Similar situation was observed in Poland, where studies encompassing years 2004–2014 revealed the presence of five subtypes of BVDV-1: 1b, 1d, 1f, 1 g [17] and 1e [18] in decreasing frequency. Later, BVDV-2a has been identified but only on one farm [19]. The aim of this study was to genotype BVDV isolates currently circulating in Poland. Such studies are important to understand epidemiology of the virus and they may support the development of successful control and eradication programs, where effective vaccines and reliable diagnostic tests are essential.

## Results

**Fig. 1:**
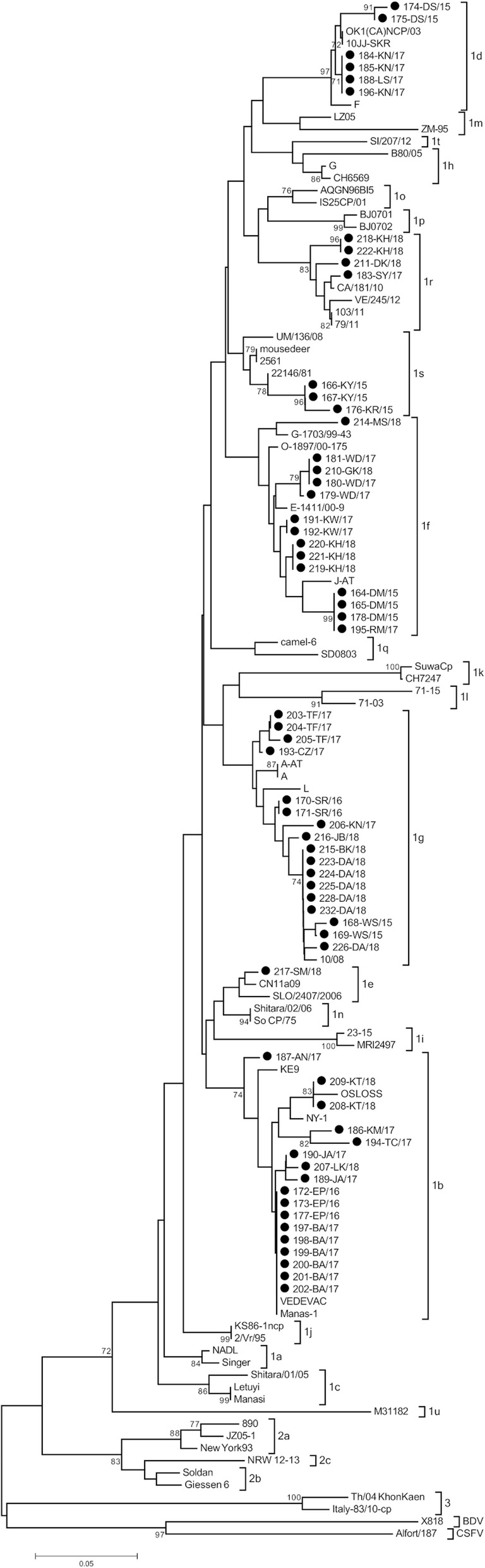
Phylogenetic tree based on 5’UTR fragment of 62 field isolates of BVDV. Black dots indicate field strains

**Fig. 2:**
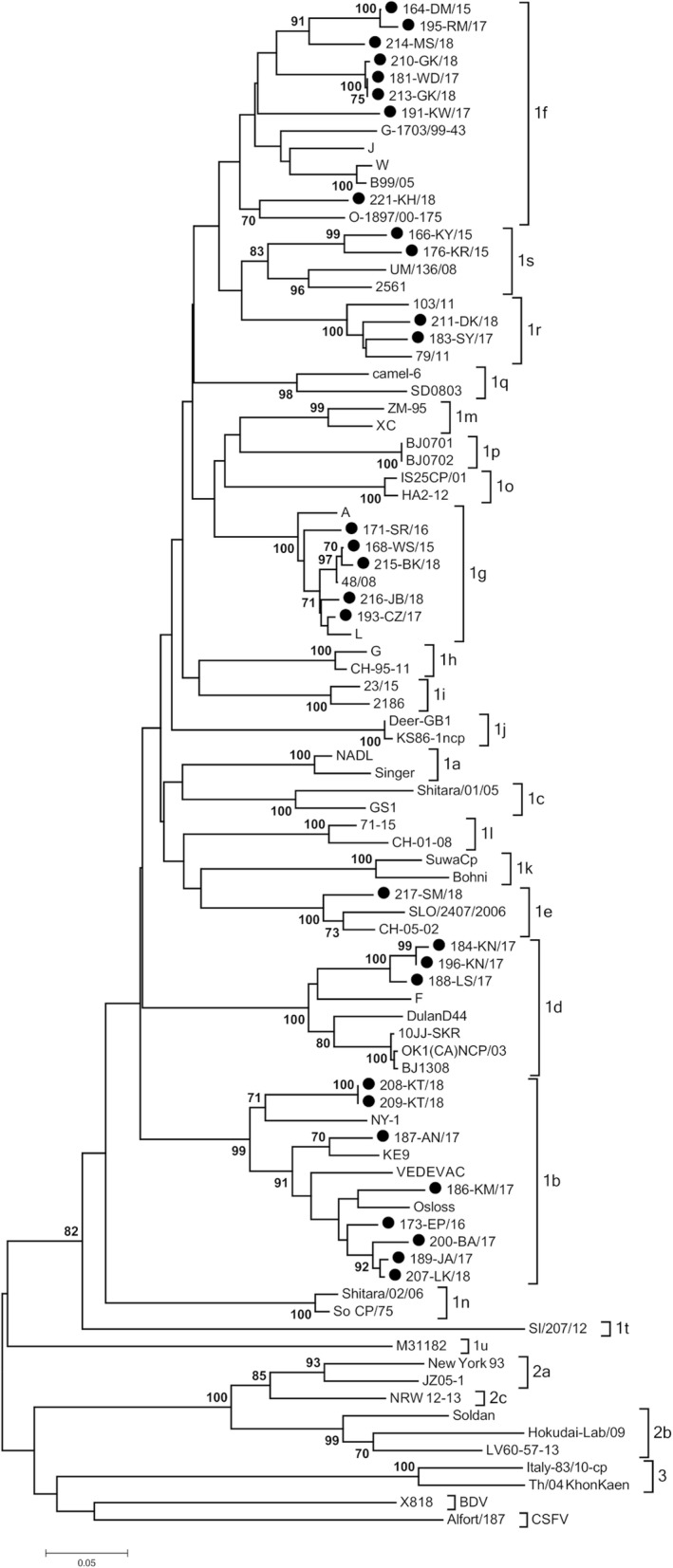
Phylogenetic tree based on N^pro^ fragment of 29 field isolates of BVDV. Black dots indicate field strains

**Fig. 3:**
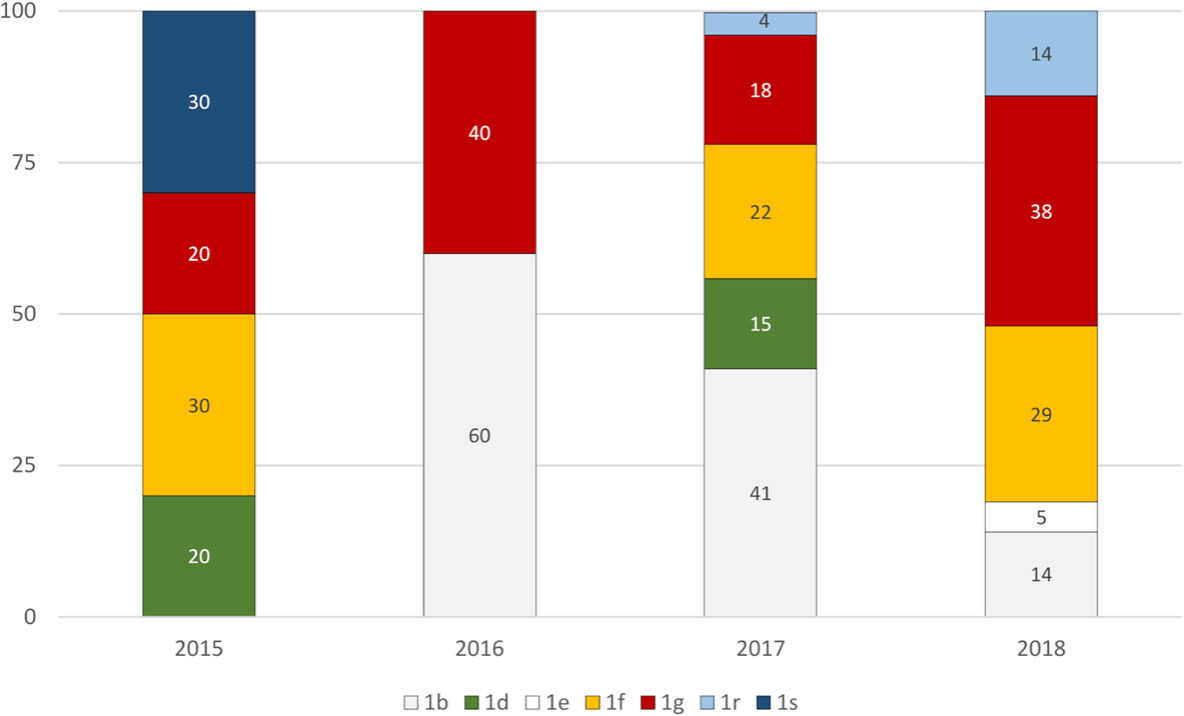
Distribution of BVDV subtypes in Poland between 2015 and 2018 (percentages)

Positive results in RT-PCR test for BVDV were obtained for 63 samples from 30 farms in all 8 provinces tested (overall prevalence of 0.7%). Nucleotide alignment with the reference strains from GenBank using BLAST tool (https://blast.ncbi.nlm.nih.gov/Blast.cgi) showed that all detected strains were characterized as BVDV-1. For phylogenetic tree construction, a 208 nucleotide fragment of the 5′UTR was analyzed and final result with the genetic relatedness of field and reference strains is shown in Fig. 1. One isolate (213-GK/18) was sequenced only in the N^pro^ region (subtype 1f) therefore, sequence analysis in the 5′ untranslated region was based on 62 sequences. Field isolates were separated into seven groups representing seven separate subtypes. Twenty nine isolates were also genotyped within N^pro^ region. The phylogenetic tree of the N^pro^ was constructed based on a 281 nucleotide fragment (Fig. 2) fully confirming classification from 5’UTR even with higher bootstrap values. Analysis revealed that BVDV-1 strains belonged to subtypes 1b detected in 8 herds (*n* = 17), 1 g in 8 herds (n = 17), 1f in 7 herds (*n* = 15), 1d in 3 herds (*n* = 6), 1r in 3 herds (r = 4), 1 s in 2 herds (*n* = 3) and 1e detected in one herd (n = 1). In order to confirm the allocation of isolates to particular subtypes another tree was constructed using the Bayesian method (Additional file 1 and Additional file 2). Field strains have been assigned to the same subtypes. The list of analyzed isolates is given in Table 1. Animals from the same herd were infected with one subtype only and sequence homology between viral isolates at herd level was very high. The only exception were two farms: one in Wielkopolskie (Farm 10) and another one in Opolskie (Farm 29) province. After initial identification of BVDV-1d (184-KN/17, 185-KN/17, 196-KN/17) in Wielkopolskie farm, another subtype, namely BVDV-1 g (206-KN/17) was identified in the same year. One year later in Opolskie province BVDV-1f was identified (219-KH/18, 220-KH/18, 221-KH/18) followed by identification for the first time in Poland of BVDV-1r (218-KH/18, 222-KH/18) in the same farm. The number of isolates per farm was between 1 and 6, although at more than 80% of farms only 1 or 2 infected individuals were identified (Table 1). The number of subtypes identified anually was 4, 2, 5 and 5 in 2015, 2016, 2017 and 2018, respectively (Fig. 3). The most predominant subtypes of BVDV-1 pear year were: 1f and 1 s (30% each) in 2015, 1b (60%) in 2016, 1b (41%) in 2017 and 1 g (38%) in 2018. The only subtype identified each year was BVDV-1 g while 1 s was identified only in 2015 (like 1e in 2018).

**Table 1:** List of field isolates used in the study

Isolate	Year of islolation	Farm	Sample	Region of isolation	Subtype	Accesion numer	
						5’UTR	N^pro^
164-DM/15	2015	1	Serum	Lublin Voivodeship	1f	MK044822	MK381419
165-DM/15	2015	1	Serum	Lublin Voivodeship	1f	MK044823	–
166-KY/15	2015	2	Serum	Kuyavian-Pomeranian Voivodeship	1 s	MK044824	MK381420
167-KY/15	2015	2	Serum	Kuyavian-Pomeranian Voivodeship	1 s	MK044825	–
168-WS/15	2015	3	Serum	Wielkopolska Voivodeship	1 g	MK044826	MK381421
169-WS/15	2015	3	Serum	Wielkopolska Voivodeship	1 g	MK044827	–
170-SR/16	2016	4	Serum	Wielkopolska Voivodeship	1 g	MK168328	–
171-SR/16	2016	4	Serum	Wielkopolska Voivodeship	1 g	MK168329	MK381422
172-EP/16	2016	5	Serum	Lublin Voivodeship	1b	MK168330	–
173-EP/16	2016	5	Serum	Lublin Voivodeship	1b	MK168331	MK381423
174-DS/15	2015	6	Serum	Wielkopolska Voivodeship	1d	MK168332	–
175-DS/15	2015	6	Serum	Wielkopolska Voivodeship	1d	MK168333	–
176-KR/15	2015	7	Serum	Kuyavian-Pomeranian Voivodeship	1 s	MK168334	MK381424
177-EP/16	2016	5	Serum	Lublin Voivodeship	1b	MK168335	–
178-DM/15	2015	1	Serum	Lublin Voivodeship	1f	MK168336	–
179-WD/17	2017	8	Serum	Lublin Voivodeship	1f	MK381356	–
180-WD/17	2017	8	Serum	Lublin Voivodeship	1f	MK381357	–
181-WD/17	2017	8	Serum	Lublin Voivodeship	1f	MK381358	MK381425
183-SY/17	2017	9	Serum	Świętokrzyskie Voivodeship	1r	MK381359	MK381426
184-KN/17	2017	10	Serum	Wielkopolska Voivodeship	1d	MK381360	MK381427
185-KN/17	2017	10	Serum	Wielkopolska Voivodeship	1d	MK381361	–
186-KM/17	2017	11	Serum	Wielkopolska Voivodeship	1b	MK381362	MK381428
187-AN/17	2017	12	Serum	Wielkopolska Voivodeship	1b	MK381363	MK381429
188-LS/17	2017	13	Serum	Wielkopolska Voivodeship	1d	MK381364	MK381430
189-JA/17	2017	14	Serum	Wielkopolska Voivodeship	1b	MK381365	MK381431
190-JA/17	2017	14	Serum	Wielkopolska Voivodeship	1b	MK381366	–
191-KW/17	2017	15	Serum	Łódź Voivodeship	1f	MK381367	MK381432
192-KW/17	2017	15	Serum	Łódź Voivodeship	1f	MK381368	–
193-CZ/17	2017	16	Serum	Wielkopolska Voivodeship	1 g	MK381369	MK381433
194-TC/17	2017	17	Serum	Wielkopolska Voivodeship	1b	MK381370	–
195-RM/17	2017	18	Serum	Kuyavian-Pomeranian Voivodeship	1f	MK381371	MK381434
196-KN/17	2017	10	Serum	Wielkopolska Voivodeship	1d	MK381372	MK381435
197-BA/17	2017	19	Serum	Wielkopolska Voivodeship	1b	MK381373	–
198-BA/17	2017	19	Serum	Wielkopolska Voivodeship	1b	MK381374	–
199-BA/17	2017	19	Serum	Wielkopolska Voivodeship	1b	MK381375	–
200-BA/17	2017	19	Serum	Wielkopolska Voivodeship	1b	MK381376	MK381436
201-BA/17	2017	19	Serum	Wielkopolska Voivodeship	1b	MK381377	–
202-BA/17	2017	19	Serum	Wielkopolska Voivodeship	1b	MK381378	–
203-TF/17	2017	20	Serum	Mazovian Voivodeship	1 g	MK381379	–
204-TF/17	2017	20	Serum	Mazovian Voivodeship	1 g	MK381380	–
205-TF/17	2017	20	Serum	Mazovian Voivodeship	1 g	MK381381	–
206-KN/17	2017	10	Serum	Wielkopolska Voivodeship	1 g	MK381382	–
207-LK/18	2018	21	Serum	Wielkopolska Voivodeship	1b	MK381383	MK381437
208-KT/18	2018	22	Serum	Lublin Voivodeship	1b	MK381384	MK381438
209-KT/18	2018	22	Serum	Lublin Voivodeship	1b	MK381385	MK381439
210-GK/18	2018	23	Serum	Lublin Voivodeship	1f	MK381386	MK381440
211-DK/18	2018	24	Lung	Mazovian Voivodeship	1r	MK381387	MK381441
213-GK/18	2018	23	Serum	Lublin Voivodeship	1f	–	MK381442
214-MS/18	2018	25	Serum	Wielkopolska Voivodeship	1f	MK381388	MK381443
215-BK/18	2018	26	Serum	Świętokrzyskie Voivodeship	1 g	MK381389	MK381444
216-JB/18	2018	27	Serum	Wielkopolska Voivodeship	1 g	MK381390	MK381445
217-SM/18	2018	28	Serum	Podlaskie Voivodeship	1e	MK381391	MK381446
218-KH/18	2018	29	Ear notch	Opole Voivodeship	1r	MK381392	–
219-KH/18	2018	29	Ear notch	Opole Voivodeship	1f	MK381393	–
220-KH/18	2018	29	Ear notch	Opole Voivodeship	1f	MK381394	–
221-KH/18	2018	29	Ear notch	Opole Voivodeship	1f	MK381395	MK381447
222-KH/18	2018	29	Ear notch	Opole Voivodeship	1r	MK381396	–
223-DA/18	2018	30	Ear notch	Wielkopolska Voivodeship	1 g	MK381397	–
224-DA/18	2018	30	Ear notch	Wielkopolska Voivodeship	1 g	MK381398	–
225-DA/18	2018	30	Ear notch	Wielkopolska Voivodeship	1 g	MK381399	–
226-DA/18	2018	30	Ear notch	Wielkopolska Voivodeship	1 g	MK381400	–
228-DA/18	2018	30	Ear notch	Wielkopolska Voivodeship	1 g	MK381401	–
232-DA/18	2018	30	Ear notch	Wielkopolska Voivodeship	1 g	MK381402	–

**Fig. 4:**
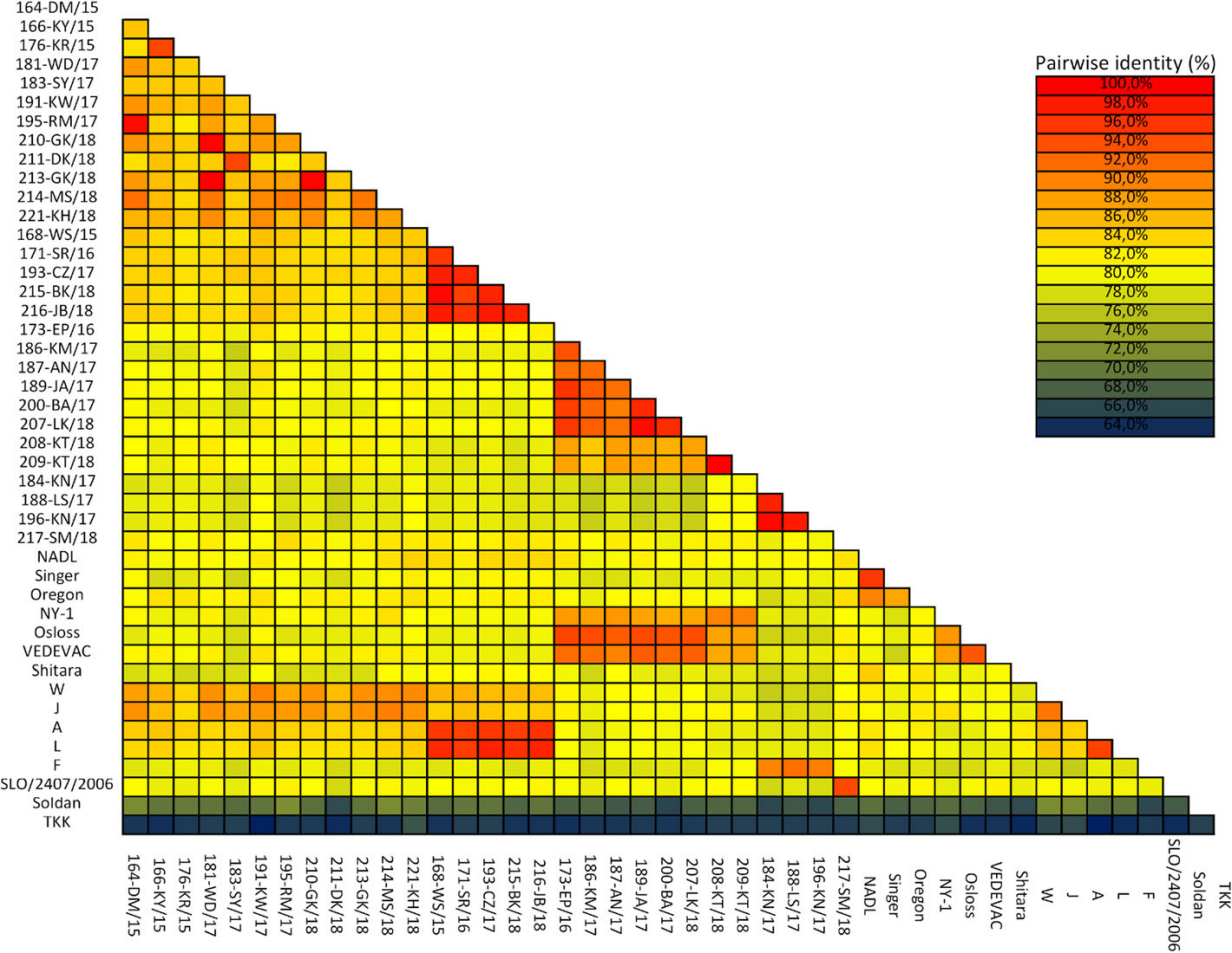
Matrix of pairwise identity scores generated by alignment of a 371 bp fragment of the N^pro^ gene for 29 Polish isolates and 15 reference strains of BVDV

Geographical clustering was observed for subtypes 1d, 1 s and 1e identified in different, single provinces. BVDV-1f was identified in 5 provinces, BVDV-1 g and BVDV-1r in 3 provinces, BVDV-1b in 2 provinces. The highest number of isolates (32) and subtypes (4), was identified in Wielkopolskie with the predominance of BVDV-1 g (41%) and BVDV-1b (37%). Second province with the highest number of positive results was Lubelskie, where 8 isolates of BVDV-1f and 5 of BVDV-1b subtypes were found. Only in two provinces (Podlaskie and Lodzkie), where positive results were obtained, single subtypes were identified. Sequence similarity between various subtypes in 5’UTR ranged from 81 to 93%. The identity percentages within same subtypes 1b, 1 g, 1f, 1d, 1r and 1 s were 91.5–100%, 96.5–100%, 91.4–100%, 92.6–100%, 96.5–98%, 99–100% respectively. Sequence similarity between various subtypes in N^pro^ region ranged from 76.5 to 86.5%. The most diverse sequences within the same subtype in N^pro^ region were identified for BVDV-1b with sequence identity values up to 84.9%. The biggest difference in subtype sequences occurred between BVDV-1b and BVDV-1d, while the tiniest variation was observed between BVDV-1f and BVDV-1 s (Fig. 4).

Sequence identity at the amino acid level in N^pro^ region among isolates tested was 78.8–100% and between various subtypes ranged from 78.8 to 93.4%. The biggest differences were observed between BVDV-1d and BVDV-1r and the smallest one between BVDV-1f and BVDV-1 g and also between BVDV-1f and BVDV-1 s. Nucleotide sequences of the BVDV strains have been submitted to GenBank with the following accession numbers: MK044822-MK044827, MK168328-MK168336, MK381356-MK381402 for 5’UTR and MK381419-MK381447 for N^pro^ region.

## Discussion

In this study, we investigated the genetic diversity of BVDV isolates from Polish herds collected between 2015 and 2018. PCR amplified sequences were subjected to sequence-based genotyping in 5′ untranslated region. The N^pro^ phylogenetic analysis confirmed typing results obtained for the 5’UTR. Viral isolates were assigned to seven subtypes in descending order of frequency of appearance: 1b, 1 g, 1f, 1d, 1r, 1 s and 1e. Previous study from years 2004–2011 described the circulation of four subtypes of BVDV-1 in Poland (1b, 1d, 1f, 1 g) with predominance of BVDV-1b and BVDV-1d [17]. In later studies, subtype 1e was also detected [18]. Current phylogenetic studies indicate that the number of BVDV subtypes has increased, however BVDV-1b is still the most often detected subtype. It is the most frequently reported subtype of BVDV worldwide. BVDV-1b is predominant in both Americas, Asia and Europe [16]. A large number of isolates belonging to subtype 1f and some of 1 g have been detected in Austria [20] and Italy [21, 22]. BVDV-1f is the most common subtype in Germany and Slovenia [16, 23]. Several studies indicate that 1f and 1 g subtypes may be unique for Europe. Viruses of BVD-1 g subtype were isolated more frequently now than in the previous study where BVDV-1 g was identified only in two herds [17]. Subtype 1d was predominant in Sweden, in years 2002–2004, when the eradication program was implemented [24]. Strains 183-SY/17, 211-DK/18, 218-KH/18 and 222-KH/18 clustered together with Italian strains belonging to subtype 1r [22]. Three strains (166-KY/15, 167-KY/15, 176-KR/15) form one clade with strains previously identified as 1f (22,146/81) [25] and 1f-like (mousedeer) [26]. Currently, together with the reference strains from Italy [22], they form the 1 s subtype [27]. BVDV-1e represented by strain 217-SM/18 has been identified only in one Polish herd. It had 98% nucleotide similarity to the Italian BVDV-1e strain from Northern Italy [28]. This subtype was found also in Switzerland [29] and France [30]. The results of this study show that the genetic heterogeneity of BVDV viruses infecting cattle in Poland has changed. These differences in subtype distribution in comparison to study from years 2004–2011 could be a result of immune selection due to natural infections and also vaccinations, which became very popular in recent years. In the present work, the evidence for geographical clustering of BVDV subtypes was not clear, unlike Italy, where BVDV-1f was predominant in northern Italy while BVDV-1b was the most frequent subtype in southern part of the country [21, 31].

HoBi-like pestiviruses (BVDV-3) do not seem to circulate in Polish cattle and BVDV-2 was found previously only in one herd [19]. BVDV-2 was first identified in North America and was associated with very high mortalities [32] from where the virus was introduced to the European continent [33]. BVDV-2 was also identified in Europe in several countries like: Italy [14], Germany [34] and Austria [20]. So far, natural infections with BVDV-3 in Europe were identified only in Italy [3]. There are suspicions that the virus has been introduced to the European continent through vaccines or other products which were prepared using contaminated bovine serum. The closest genetically related strains to Polish isolates were identified in Slovenia, United Kingdom and Italy according to blastn analysis. High level of similarity among these viruses may suggest a common ancestor.

Only a few inactivated and recently also modified-live vaccines are commercially available in Poland. In this study BVDV was identified in 6 herds from animals previously vaccinated with killed vaccines. Three herds were infected with BVDV-1b subtype (strains 187-AN/17, 189-JA/17, 190-JA/17 and 194-TC/17), two with BVDV-1d (184-KN/17, 185-KN/17, 188-LS/17) and one herd was infected with BVDV-1 g (215-BK/18). In all these herds protective vaccinations were based on BVDV-1a strain, and they were introduced after PIs removal. Interestingly subtype 1a has never been identified in Poland, which could be the effect of selection force induced by vaccines based on this subtype. Other studies have shown significant differences in antibody levels in serum from calves receiving modified live virus vaccines based on BVDV-1a, with a significantly lower BVDV-1b antibody titres [35]. PI individuals infected with BVDV-1b were identified in one Polish herd vaccinated with a killed vaccine based on BVDV-1a [36]. Although clinical symptoms resembling BVD were not observed in that herd, the protection offered by vaccinal strain did not provide cross protection against BVDV-1b. Vaccination strategy should take into consideration both genetic and antigenic diversity of the virus present in the region where vaccination is implemented and therefore, effective vaccine should include the subtypes of local isolates. For this reason monitoring of newly emerging strains is important for successful control and eradication programs and it requires constant updates. Antigenic differences among individual subtypes of BVDV-1 occur as well [37]. Therefore, more cross-protection studies should be carried out to address the importance of this diversity. It seems reasonable to include a mixture of several viral subtypes present in local herds when designing effective vaccines. Phylogenetic studies with increasing cattle trade can also help to identify potential sources and routes of virus introduction, although such sources were not identified for Polish isolates, probably due to significant diversity of the virus in every country studied.

The genetic diversity is also important for laboratory diagnosis, since it can hamper the ability of diagnostic methods to identify as many viral subtypes as possible. In this study we used specific primers for non-coding 5’UTR and coding N^pro^ region. 5’UTR is highly conserved among the pestiviruses. It contains *cis*-acting elements required for viral replication and translation [38]. N^pro^ (N-terminal protein) of BVDV encodes for a cysteine protease that cleaves the N-terminus from the core protein. N^pro^ also prevents interferon-α/β induction in infected cells [39]. The validity of 5’UTR classification in this study was confirmed by the parallel analysis of N^pro^ sequences. RT-PCR used in this study [40], which is commonly used for BVDV detection, does not detect or detects with low efficiency strains of HoBi-like viruses due to the presence of a mismatch at the 3′ end of the forward primer which does not allow proper annealing [41]. This disadvantage may lead to false negative results when testing field samples for BVDV-3 and therefore we implemented real-time PCR enabling the detection of all three species of BVDV with high sensitivity. This new method was implemented to study doubtful PCR results although all samples turned negative when tested with real-time PCR.

## Conclusion

In summary, the distribution of subtypes in Poland has changed. Two new subtypes 1r and 1 s were detected for the first time. Monitoring of strains circulating in a given country is a useful indicator in the aspect of designing an effective vaccination program or a reliable diagnostic test.

## Methods

### Sample collection

A total of 9290 serum, tissue homogenate, ear notch and semen samples were collected in years 2015–2018. The animals used in the study came from private farms, where infection with BVDV was suspected based on clinical symptoms or where eradication was under way. The owners of those herds provided local vets with their permissions to collect samples for laboratory testing. Samples were collected in 8 out of 16 provinces of Poland: Kujawsko-Pomorskie, Lubelskie, Łódzkie, Opolskie, Świętokrzyskie, Mazowieckie, Wielkopolskie and Podlaskie. Cattle population in last three provinces comprises 51% of the total population of this ruminant species in Poland. For comparison studies sequences of 81 reference strains of different species and subtypes of BVDV and single strains of BDV and CSFV were retrieved from GenBank (Table 2).

**Table 2:** List of reference strains used for phylogenetic comparison with Polish isolates

Pestivirus species	Subtype	Strain	5’UTR Accesion number	N^pro^ Accesion number
BVDV-1	1a	NADL	AJ133738	AJ133738
BVDV-1	1a	Singer	DQ088995	DQ088995
BVDV-1	1b	VEDEVAC	AJ585412	AJ585412
BVDV-1	1b	OSLOSS	AY279528	M96687
BVDV-1	1b	Manas-1	EU555288	–
BVDV-1	1b	New York-1 (NY-1)	FJ387232	FJ387232
BVDV-1	1b	KE9	EF101530	EF101530
BVDV-1	1c	Shitara/01/05	AB359926	AB359926
BVDV-1	1c	GS1	–	JQ071526
BVDV-1	1c	Letuyi	EU159701	–
BVDV-1	1c	Manasi	EU159702	–
BVDV-1	1d	F	AF298065	AF287284
BVDV-1	1d	OK1(CA)NCP/03	AB359927	AB359927
BVDV-1	1d	DulanD44	–	KC414609
BVDV-1	1d	10JJ-SKR	KC757383	KC757383
BVDV-1	1d	BJ1308	–	KT951841
BVDV-1	1e	SLO/2407/2006	KX577637	KX577637
BVDV-1	1e	CN11a@09	MG434588	–
BVDV-1	1e	CH-05-02	–	EU180036
BVDV-1	1f	J-AT	FJ493480	–
BVDV-1	1f	J	–	AF287286
BVDV-1	1f	W	–	AF287290
BVDV-1	1f	O-1897/00–175	AY323895	AY323895
BVDV-1	1f	G-1703/99–43	AY323876	AY323876
BVDV-1	1f	E-1411/00–9	AY323872	–
BVDV-1	1f	B99/05	–	EU224259
BVDV-1	1 g	L	FJ493483	AF287287
BVDV-1	1 g	A-AT	FJ493482	–
BVDV-1	1 g	A	AF298064	AF287283
BVDV-1	1 g	10/08	JN715004	–
BVDV-1	1 g	48/08	–	JN833739
BVDV-1	1 h	G	AF298066	AF287285
BVDV-1	1 h	CH6569	MH907191	–
BVDV-1	1 h	B80/05	EU224239	–
BVDV-1	1 h	CH-95-11	–	EU180042
BVDV-1	1i	23–15	AF298059	AF287279
BVDV-1	1i	2186	–	JQ920329
BVDV-1	1i	MRI2497	LT902628	–
BVDV-1	1j	KS86-1ncp	AB078950	AB078950
BVDV-1	1j	2/Vr/95	AJ293594	–
BVDV-1	1j	Deer-GB1	–	U80902
BVDV-1	1 k	SuwaCp	AF117699	AY894998
BVDV-1	1 k	CH7247	MH907869	–
BVDV-1	1 k	Bohni	–	AY894997
BVDV-1	1 l	71–03	KF205294	–
BVDV-1	1 l	71–15	KF205306	KF205329
BVDV-1	1 l	CH-01-08	–	EU180033
BVDV-1	1 m	LZ05	GU120241	–
BVDV-1	1 m	ZM-95	AF526381	AF526381
BVDV-1	1 m	XC	–	MH166806
BVDV-1	1n	Shitara/02/06	AB359930	AB359930
BVDV-1	1n	So CP/75	AB359929	AB359929
BVDV-1	1o	AQGN96BI5	AB300691	–
BVDV-1	1o	IS25CP/01	AB359931	AB359931
BVDV-1	1o	HA2–12	–	KX218370
BVDV-1	1p	BJ0701	GU120247	GU120259
BVDV-1	1p	BJ0702	GU120248	GU120260
BVDV-1	1q	camel-6	KC695810	KC695810
BVDV-1	1q	SD0803	JN400273	JN400273
BVDV-1	1r	VE/245/12	LM994671	–
BVDV-1	1r	CA/181/10	LM994672	–
BVDV-1	1r	79/11	KY040384	KY040432
BVDV-1	1r	103/11	KY040372	KY040425
BVDV-1	1 s	UM/136/08	LM994673	LN515612
BVDV-1	1 s	mousedeer	AY158154	–
BVDV-1	1 s	2561	JQ920287	JQ920343
BVDV-1	1 s	22,146/81	AJ304376	–
BVDV-1	1 t	SI/207/12	LM994674	LN515611
BVDV-1	1u	M31182	JQ799141	JQ799141
BVDV-2	2a	New York’93	AF502399	KR093034
BVDV-2	2a	890	L32886	–
BVDV-2	2a	JZ05–1	GQ888686	GQ888686
BVDV-2	2b	Soldan	U94914	AY735495
BVDV-2	2b	Giessen 6	AY379547	–
BVDV-2	2b	Hokudai-Lab/09	–	AB567658
BVDV-2	2b	LV60–57-13	–	KM217405
BVDV-2	2c	NRW 12–13	HG426483	HG426483
BVDV-3	3	Th/04_KhonKaen (TKK)	FJ040215	FJ040215
BVDV-3	3	Italy-83/10-cp	JQ612705	JQ612705
BDV	–	X818	AF037405	AF037405
CSFV	–	Alfort/187	NC 038912	NC 038912

### RNA extraction and RT-PCR

Total RNA was extracted using TRI Reagent (Sigma-Aldrich, USA) from 500 μl of serum, tissue homogenates, cell culture medium after overnight soaking of ear notches or from diluted semen following the manufacturer’s instructions and stored at -80 °C until testing. Reverse transcription-polymerase chain reaction (RT-PCR) was carried out using the Transcriptor One-Step RT-PCR Kit (Roche) in a 25 μl reaction mix consisting of PCR buffer 5 μl, water DEPC 15.5 μl, set of primers 1 μl (10 μM), 0.5 μl enzyme mix and 2 μl of template RNA. Reverse transcription was performed at 50 °C for 30 min using reverse primer. cDNA was amplified using primers pair specific for BVDV 5′ untranslated region: 324F (5′-ATGCCCWTAGTAGGACTAGCA-3′) and 326R (5′-TCAACTCCATGT GCCATGTAC-3′) [40]. PCR thermal conditions were the following: initial denaturation at 94 °C for 7 min followed by 35 cycles of denaturation at 94 °C for 10 s, primer annealing at 53 °C for 30 s and elongation at 68 °C for 30 s. The final elongation was extended to 7 min at 68 °C. Primers specific for N^pro^ region: B32-F (TGCTACTAAAAATCTCTGCTGT) and B31-R (CCATCTATrCAyACATArATGTGGT) [23] were used with thermal profile of 94 °C for 15 s, 50 °C for 30s and 68 °C for 1 min for 35 cycles and 10 min in 68 °C for final elongation. Approximate sizes of PCR products were 288 bp and 441 bp for 5’UTR and N^pro^ region respectively.

### Sequencing and phylogenetic analysis

The PCR products were sequenced in both directions with the same primers used for amplification using Big Dye Terminator v3.1 Cycle Sequencing Kit with a 3730XL Genetic Analyzer (Applied Biosystems). The DNA fragments were purified using a QIAquick PCR Purification kit (Qiagen), following the analysis in a 16-capillary sequencer ABI PRISM 3100 Genetic Analyzer (Applied Biosystems). The consensus of each genetic region was determined by the alignment of forward and reverse strand sequences using Clustal Omega tool of the European Molecular Biology Laboratory (http://www.ebi.ac.uk). Sequences generated in this study were aligned with the analogous sequences of reference pestivirus strains deposited in the GenBank database (Table 2) using the ClustalW algorithm from Molecular Evolutionary Genetics Analysis software package, version 5.2 (MEGA 5.2). Phylogenetic trees were constructed using neighbor-joining algorithm [42] with a Kimura 2-parameter substitution model [43] with 1000 bootstrap replicates. Phylogenetic trees were also constructed by the Bayes method with the GTR substitution model using the tree-builder tool of the Geneious software [44]. Sequence identity (%) among strains was calculated using the identity matrix in BioEdit v.7.2.5 software [45].

## Additional files

Additional file 1: Phylogenetic relationship between field and reference strains inferred by Bayesian analysis in 5’UTR. The figure shows a phylogenetic tree created on the basis of the 5’UTR fragment by the Bayes method with the GTR substitution model. It consists of 62 field isolates and representatives of all known subtypes of the BVDV-1 species, representatives of the BVDV-2, BDV and CSFV species. (PDF 148 kb)

Additional file 2: Phylogenetic relationship between field and reference strains inferred by Bayesian analysis in N^pro^ region. The figure shows a phylogenetic tree created on the basis of the fragment of the Npro region by the Bayes method with the GTR substitution model. It consists of 29 field isolates and representatives of all known subtypes of the BVDV-1 species, representatives of the BVDV-2, BDV and CSFV species. (PDF 120 kb)

## Data Availability

The data sets supporting the results of this article are included within the article.

## Abbreviations

5’UTR: Untranslated region 5′
BVDV: Bovine viral diarrhea virus
N^pro^: N-terminal protease
PCR: Polymerase chain reaction
PI: persistently infected
RT: Reverse transcription
