# Supplementary figures and images for: Increased genetic variation of bovine viral diarrhea virus in dairy cattle in Poland
Source: BMC Vet Res. 2019 Aug 5;15:278. doi: 10.1186/s12917-019-2029-z (PMC6683398; doi:10.1186/s12917-019-2029-z)

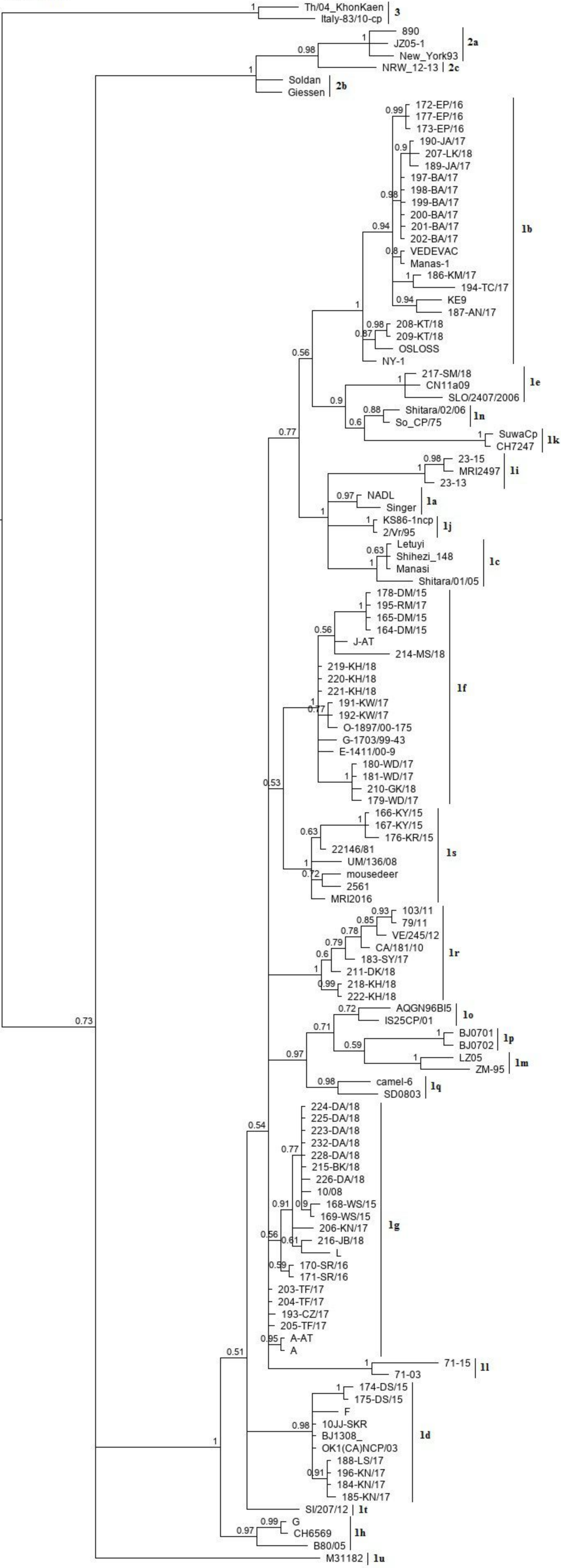

Supplement: Supplementary file 1 — Phylogenetic relationship between field and reference strains inferred by Bayesian analysis in 5’UTR. The figure shows a phylogenetic tree created on the basis of the 5’UTR fragment by the Bayes method with the GTR substitution model. It consists of 62 field isolates and representatives of all known subtypes of the BVDV-1 species, representatives of the BVDV-2, BDV and CSFV species. (PDF 148 kb) [file 12917_2019_2029_MOESM1_ESM.pdf]

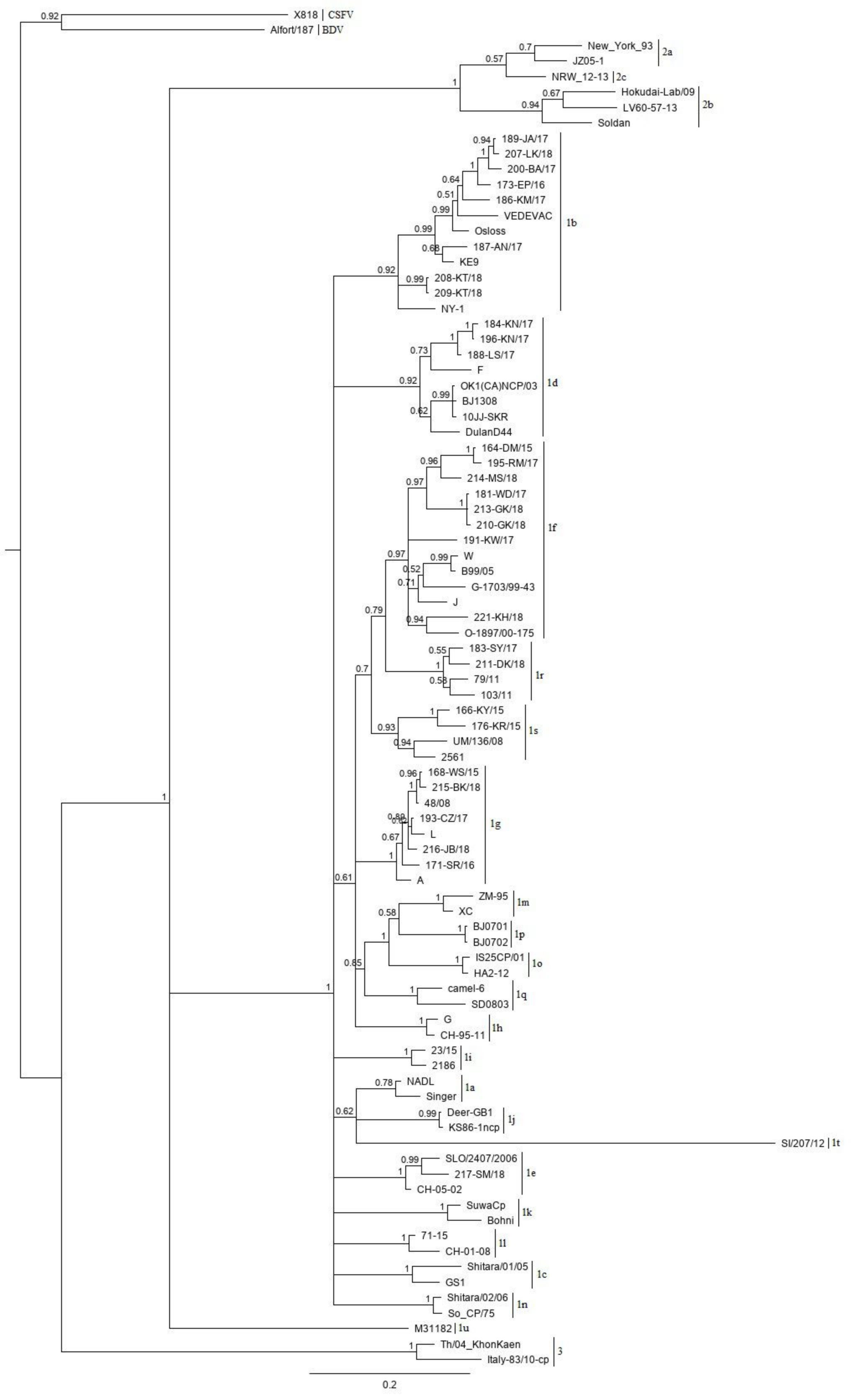

Supplement: Supplementary file 2 — Phylogenetic relationship between field and reference strains inferred by Bayesian analysis in Npro region. The figure shows a phylogenetic tree created on the basis of the fragment of the Npro region by the Bayes method with the GTR substitution model. It consists of 29 field isolates and representatives of all known subtypes of the BVDV-1 species, representatives of the BVDV-2, BDV and CSFV species. (PDF 120 kb) [file 12917_2019_2029_MOESM2_ESM.pdf]
